# Structured higher protein meal replacement plans for weight loss and metabolic health: A qualitative narrative evidence review of Medifast interventions

**DOI:** 10.1016/j.obpill.2026.100265

**Published:** 2026-04-08

**Authors:** Alexandra J. Miller, Christopher D. Coleman, Jessica R. Kiel, Kristen M. Beavers, Satya S. Jonnalagadda

**Affiliations:** a Department of Scientific and Clinical Affairs, Medifast, Inc., 1501 S Clinton St, Ste 500, Baltimore, MD, 21224, USA; b Department of Scientific and Clinical Affairs, Medifast, Inc., 11515 Cronridge Drive, Owings Mills, MD, 21117, USA; c Department of Internal Medicine, Section of Gerontology and Geriatric Medicine, Wake Forest University School of Medicine, Medical Center Boulevard, Winston-Salem, NC, 27157, USA

**Keywords:** Fat mass, High protein diet, Lean mass, Meal replacement, Metabolic health, Weight loss

## Abstract

**Background:**

Excess body weight and impaired metabolic health increase chronic disease risk, necessitating effective weight management strategies. Commercial weight loss programs are widely used, but impact on body composition and cardiometabolic markers requires evaluation. The objective of this narrative review was to assess the efficacy of Medifast's structured higher protein meal replacement plans with comprehensive lifestyle intervention.

**Methods:**

In this narrative qualitative evidence review, publications (2010–2025) utilizing Medifast meal plans (800–1000/80–120; 1100–1300/120–150; 1300–1500/140–170 kcal/g protein/day) as a weight loss intervention were identified*.* Two reviewers extracted data on study design, population characteristics, interventions, outcomes and corresponding results, as reported in the publications; a third verified accuracy.

**Results:**

Seventeen publications representing nine unique randomized controlled trials (RCTs) and additional secondary analyses and chart reviews were included (n = 20–816; 4–26 weeks duration). Participants were adults living with overweight/obesity, including older adults and those with coexisting conditions (e.g., post-prostatectomy, post-ischemic stroke). Thirteen of 14 studies reported clinically meaningful weight loss (≥5%) with 7 reporting ≥10%. Total fat mass (FM) was significantly reduced in all reporting studies (3.2–11.0 kg; 11/11 studies; *p* < 0.05) and visceral FM in 4/5 studies (varied reporting; *p* < 0.05), while lean mass was preserved within ≤6% baseline (13/13 studies). Favorable decreases occurred in blood pressure (3.2–19.6 mmHg SBP; 1.6–9.0 mmHg DBP); significance varied. Outcomes for lipid and glycemic markers were mixed; significant between-group improvements (*p* < 0.05) were observed for total and LDL cholesterol (2/5 RCTs), fasting insulin (3/4 RCTs), and A1c (1/1 RCT).

**Conclusions:**

Medifast's structured, higher protein meal replacement plans with comprehensive lifestyle interventions yield clinically meaningful short-term weight loss and favorable body composition changes; cardiometabolic outcomes vary. While long-term durability data are needed, clinicians may consider these plans as options for the dietary foundation within the obesity care framework, tailored to individual patient needs.

## Graphical abstract

**Figure:**
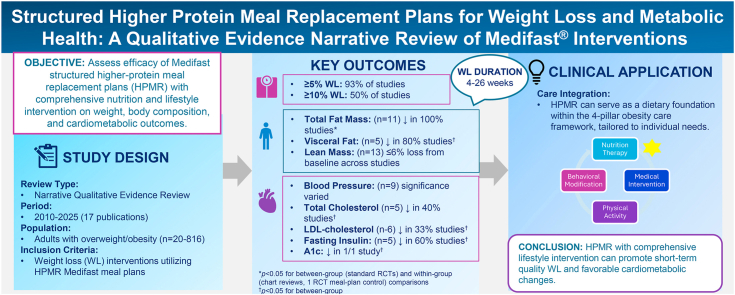

## 1 Introduction

Metabolic health encompasses the ability to maintain favorable levels of adiposity, blood glucose, blood pressure, lipid profiles and overall energy regulation. Metabolic dysfunction represents a continuum of abnormalities including metabolic syndrome and broader cardiometabolic irregularities that increase risk of type 2 diabetes, cardiovascular disease, and other chronic conditions [[1], [2], [3]]. Recent data indicate only 6.8% of U.S. adults have optimal cardiometabolic health, with significant declines over the past two decades [3].

Weight reduction of 5%–7% is associated with improvements in glycemic control, blood pressure, and lipid profiles in adults with overweight or obesity [4,5]. While pharmacologic and surgical approaches are increasingly used, lifestyle interventions remain a cornerstone of weight management [6,7]. Calorie-restricted dietary approaches can be effective when nutritionally adequate and sustainable [4,5].

In addition to weight reduction, improving key cardiometabolic risk factors, including central adiposity, glycemic control, blood pressure, and blood lipids, is central to improving metabolic health and lowering the risk of downstream chronic disease [1,4,6,[8], [9], [10]]. Increasingly, attention has turned to body composition as a key mediator of cardiometabolic risk. Reducing central adiposity (e.g., waist circumference, visceral adiposity) while preserving lean, metabolically active tissue, is recognized as an important determinant of cardiometabolic health [[11], [12], [13], [14], [15]]. Indices of central obesity (e.g. waist circumference ≥102 cm for males, ≥88 cm for females) and excess visceral fat are strong predictors of metabolic syndrome, diabetes, and other cardiometabolic outcomes, beyond body mass index (BMI) alone [2,[11], [12], [13], [14], [15]].

One strategy intended to help facilitate weight loss and improve cardiometabolic risk factors is the use of structured commercial meal replacement programs. These programs are designed to provide structured nutrition and lifestyle support through portion control, reduced decision burden, micronutrient fortification during energy restriction, and convenience, along with a focus on other key behavior modification strategies [16,17]. Meal replacement approaches are cited in obesity guidelines as an effective modality for calorie-restricted weight loss [4,7,8]; however, their impact on broader metabolic health outcomes when delivered as part of a comprehensive lifestyle intervention warrants further synthesis. Clinicians may benefit from up-to-date, program-specific evidence for the commercial interventions they encounter in routine practice.

### 1.1 Objective

This narrative qualitative evidence review examined published evidence for weight loss interventions utilizing Medifast's structured, higher protein meal replacement plans (5&1®, 4&2&1®, and 5&2&2®) with comprehensive lifestyle intervention and their impact on weight and metabolic health outcomes in adults living with overweight or obesity.

## 2 Methods

### 2.1 Study selection and eligibility criteria

**Fig. 1:**
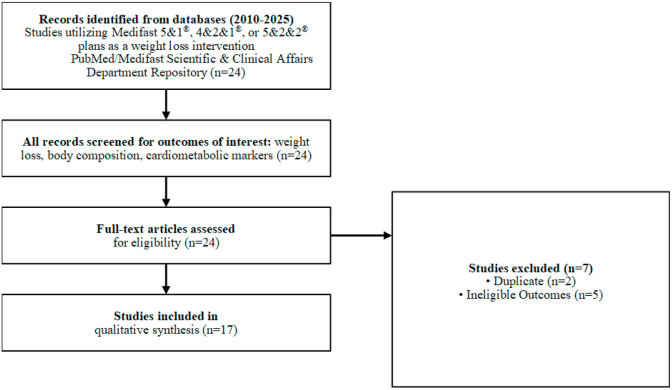
**Study Selection Diagram.** This diagram illustrates the methodological process used to identify, screen, and select publications for the narrative qualitative evidence review. From the initial 24 publications reviewed, 17 met the inclusion criteria for final synthesis.

Studies utilizing a Medifast meal plan (5&1, 4&2&1, and/or 5&2&2 Plans) as a weight loss intervention were identified from a comprehensive repository of all peer-reviewed publications and clinical trials evaluating Medifast meal plans maintained by Medifast's Scientific & Clinical Affairs department. This repository is maintained through ongoing monitoring of literature databases and clinical trial registries (e.g., PubMed, ClinicalTrials.gov) to ensure all investigator-initiated and Medifast program-sponsored research is captured. To ensure transparency and thoroughness, all identified studies meeting the specific inclusion criteria were reviewed, as summarized in the Study Selection Diagram (Fig. 1). To minimize selection bias, all identified studies meeting predefined inclusion criteria were included regardless of study design, outcomes, or publication source.

To be included in this review, publications had to meet the following criteria: (1) utilize one of Medifast's three core structured meal plans (5&1, 4&2&1, or 5&2&2), (2) report weight or metabolic health outcomes, and (3) be published in a peer-reviewed journal between January 2010 and June 2025. Publications in which Medifast meal replacements were used without one of the above meal plans, studies with only abstracts, and conference proceedings were excluded.

### 2.2 Description of Medifast meal plans

The Medifast meal plans utilized in the reviewed studies follow standardized nutritional protocols as described in previous publications [[18], [19], [20]]. These plans combine portion-controlled meal replacements (MRs), self-prepared Lean and Green meals, and, in some plans, “Healthy Snacks.” Individual MRs provide approximately 90–110 kcal, 11–15 g protein, 12–15 g carbohydrate, 4–6 g fiber, and 0–3.5 g fat. They are fortified with at least 20% of the daily value for 20 essential vitamins and minerals, providing 80%–100% of the daily value when consumed as part of Medifast's structured meal plans. Nutritionally, these meal replacements utilize high-quality protein from soy and dairy sources and contain no colors, flavors, or sweeteners from artificial sources. Lean and Green meals consist of 5–7 oz. of cooked lean protein, three servings of non-starchy vegetables, and up to two servings of healthy fats. Healthy Snacks typically consist of one serving of fruit, dairy, starch, or a prepackaged program snack. More specifically, on a daily basis, the 5&1 Plan provides 800 to 1000 kcal and 80–120 g of protein, the 4&2&1 Plan provides 1000 to 1200 kcal and 120–150 g of protein, and the 5&2&2 Plan provides 1300 to 1500 kcal and 140–170 g of protein.

While these plans are designed for weight loss, the program includes a structured transition and maintenance phase where meal replacements are gradually replaced with nutrient-dense whole foods. In the weight maintenance phase, individuals may continue using a reduced number of meal replacements as a weight management tool while continuing the healthy eating habits initiated during weight loss.

### 2.3 Behavioral and physical activity components

The Medifast program integrates structured meal plans with multimodal behavioral support, offering customizable interventions, such as coaching and comprehensive lifestyle curricula, designed to facilitate long-term habit formation. For most of the included studies, physical activity was generally encouraged but not required or standardized except for Beavers et al., 2025 [21].

### 2.4 Data extraction and study classification

Data extraction was performed using a standardized Excel template by two reviewers (one of whom is the lead author), with a third reviewer performing quality assurance on the extracted data and in the review process; two of these reviewers were independent team members. For each eligible publication, extracted data included the study design, population characteristics, intervention duration, primary and secondary outcomes and corresponding results. Because several trials (n = 6) had multiple publications, related reports were identified and grouped and treated as a single unique study to avoid duplication of outcomes.

When present, the control group for each study was classified (e.g., standard food-based calorie restriction or no structured intervention) to facilitate the interpretation of effects associated with weight loss interventions using Medifast meal plans. In some publications, like chart reviews, there was no control group and, in three studies a Medifast meal plan served as the control [[21], [22], [23]].

Two randomized controlled trials (RCTs) and two chart reviews included both weight loss and maintenance phases; for cross-study comparability, outcomes and corresponding results were extracted from the weight loss phase only [20,[24], [25], [26]]. This approach aligns with the primary design of the Medifast meal plans and was the focus of most included trials (i.e., weight loss) and the focus of this narrative qualitative evidence review.

Across the reviewed publications, several additional outcomes were reported that fell outside the predefined scope of this synthesis, which focused specifically on weight, body composition, and cardiometabolic health. For example, data were reported for inflammatory markers [24,25,[27], [28], [29], [30], [31], [32], [33]], bone biomarkers [21,32,34], quality of life [27,30,31,35], and behavioral adherence [22,24,25,35,36]. While these data were not synthesized here, they provide further context on the multi-dimensional impact of these interventions and are available in the primary and secondary reports of the included trials.

### 2.5 Computation and standardization of outcomes

For all variables, the measurement method, assessment time points, and reported results per the authors were documented. Where the original publication provided baseline and change in measurements but did not report a percent change, these were calculated by the study authors (change/baseline x100). All such calculated values are explicitly identified in the summary tables.

Given heterogeneity in study cohorts and protocols in this narrative qualitative evidence review, data were synthesized qualitatively rather than through a pooled meta-analysis. As part of the qualitative review process, statistical significance was recorded as reported by the original publication, distinguishing between-group differences and within-group changes from baseline to end of intervention, or to primary endpoint as reported by the authors.

## 3 Results

Twenty-four publications were reviewed; 17 publications were identified that fit the inclusion criteria (Fig. 1, Table 1). Within those 17 publications, there were 9 unique RCTs (n = 34–198), which were described across 10 publications (including the 9 primary reports and 1 companion article reporting additional bone and cardiometabolic outcomes (n = 96)) [37]. Among the RCTs, 7 had standard dietary controls or no dietary control (weight stable control) and 2 had Medifast meal plans as the control (weighted vest and/or resistance training were the independent variable). Additionally, the evidence base included 1 non-randomized open-label pilot intervention study (n = 20), 3 retrospective chart reviews (n = 62–446) [[18], [19], [20]], and 3 secondary data analyses (n = 58–816).

**Table 1:** **Overview of Studies**. Summary of study designs, participant characteristics, and intervention protocols for the 17 included publications.

Author, Year	Study Design	Participants/Condition/Age	Medifast Meal Plan	Control/Comparator	Study Duration	Weight Loss Duration
Beavers et al., 2025^a^	RCT	n = 150; 60–85 yrs men/women living with obesity	4&2&1	Arm 1: 4&2&1 Plan (Control)	12 months	12 months
				Arm 2: 4&2&1 Plan + Weighted Vest		
				Arm 3: 4&2&1 Plan + Resistance Training		
Bechtel et al., 2024	RCT	n = 40; men ≥50 yrs living with overweight/obesity and undergoing prostatectomy	5&2&2	Standard counseling and educational materials	4–16 weeks WL (mean = 5 weeks) + 6 months WM	4–16 weeks (mean = 5 weeks)
Justice et al., 2024	Secondary data analysis	n = 58; generally healthy sedentary men/women living with overweight/obesity and pre-diabetes	4&2&1	No dietary intervention; WS	18 weeks	18 weeks
Dearborn et al., 2023	RCT	n = 34; men/women living with overweight/obesity and recent ischemic stroke	4&2&1	No dietary intervention/standard care	12 weeks	12 weeks
Hamilton-Reeves et al., 2021	Non-randomized open-label pilot	n = 20; men 50–72 yrs living with overweight/obesity and undergoing prostatectomy	5&2&2	No dietary intervention	5–16 weeks (mean = 8.3 weeks) + 12 weeks WM	5–16 weeks (mean = 8.3 weeks)
Arterburn et al., 2019	RCT	n = 198; generally healthy men/women (18–65 yrs) living with overweight/obesity	4&2&1 (MED) 5&1 (OPT)	Self-directed, reduced calorie diet	16 weeks	16 weeks
Serra et al., 2019	Companion article to RCT (Beavers 2019)	n = 96; older (65–79 yrs) men/women living with obesity and mobility disability	4&2&1	No dietary intervention; WS	6 months	6 months
Beavers et al., 2019	RCT	n = 96; older (65–79 yrs) men/women living with obesity and self-reported mobility disability	4&2&1	No dietary intervention; WS	6 months	6 months
Normandin et al., 2018^a^	RCT	n = 37; men/women 65–79 yrs living with obesity and sedentary	4&2&1	Arm 1: 4&2&1 Plan (Control)	22 weeks	22 weeks
				Arm 2: 4&2&1 Plan + Weighted Vest		
Shaver et al., 2018	Secondary data analysis (Beavers 2019)	n = 95; older (65–79 yrs) men/women living with obesity and mobility disability	4&2&1	No dietary intervention; WS	6 months	6 months
Coleman et al., 2017	Secondary data analysis (Coleman 2015, Kiel 2015, Coleman 2012)	n = 816 charts; men/women ≥18 yrs living with overweight/obesity	5&1; 4&2&1; 5&2&2	Clients without self-reported diabetes/high blood sugar	24 weeks PE, FV varied by client WL goals	24 weeks^b^
Moldovan et al., 2016^a^	RCT	n = 77; men/women 35–70 yrs living with obesity	5&1	Arm 1: 5&1 Plan (Control)	12 weeks	12 weeks
				Arm 2: 5&1 Plan with phentermine		
Coleman et al., 2015	Retrospective chart review	n = 310; men/women ≥18 yrs living with overweight/obesity	4&2&1	n/a	12 weeks PE WL, FV varied by client goals, WM mean = 34 weeks	12 weeks^b^
Kiel et al., 2015	Retrospective chart review	n = 62; men/women ≥18 yrs living with overweight/obesity	5&2&2	n/a	12 weeks PE, FV varied by client goals, WM mean = 17 weeks	12 weeks^b^
Shikany et al., 2013	RCT	n = 120; men/women 19–65 yrs living with obesity	5&1	Reduced energy food-based diet	52 weeks (26 weeks WL, 26 weeks WM)	26 weeks
Coleman et al., 2012	Retrospective chart review	n = 446; men/women ≥18 yrs living with overweight/obesity	5&1	n/a	FV mean = 19.6 ± 13.5 weeks^b^	FV mean = 19.6 ± 13.5 weeks^b^
Davis et al., 2010	RCT	n = 90; men/women ≥18 yrs living with obesity	5&1	Reduced energy food-based diet	40 weeks (16 weeks WL, 24 weeks WM)	16 weeks

**Notes:** Overweight is defined as a body mass index (BMI) of 25.0–29.9 kg/m^2^. Obesity is defined as a BMI ≥30.0 kg/m^2^. RCT = randomized controlled trial, yrs = years, n/a = not applicable, WL = weight loss, WM = weight maintenance, WS = weight stable, PE = primary endpoint, FV = final visit.

a While this RCT included 3 arms, only results from the 4&2&1 Plan (control) arm are reported here to isolate the effects of the meal plan intervention without confounding physical activity variables.

b primary endpoint as reported by authors.

Participants in all studies included adults living with overweight or obesity and further included older adults (up to 85 years), men ≥50 years undergoing a prostatectomy, adults with a recent ischemic stroke, and generally healthy adults interested in losing weight. Study durations ranged from 4 to 26 weeks for the weight loss interventions.

### 3.1 Weight loss

Weight loss outcomes are summarized in Table 2. Thirteen of 14 studies reported clinically meaningful weight loss (≥5%) with 7 reporting ≥10%; greater weight loss was generally observed in studies of longer duration. In the reviewed RCTs, mean weight loss typically ranged from approximately 5%–12% of baseline body weight over 4–26 weeks [[21], [22], [23], [24], [25],^27^,^28^,^36^]. In retrospective chart reviews, average weight loss of approximately 9%–16% over 12–24 weeks were reported [[18], [19], [20],^26^]. In most study populations with coexisting conditions (e.g., men awaiting prostatectomy, adults with prediabetes/diabetes), similar magnitudes of weight loss were observed [26,27,29,30], whereas one pilot trial in individuals with recent ischemic stroke (n = 34) showed a modest, nonsignificant change; the authors noted a weight-gain outlier may have influenced results in this pilot study [35] (Table 2).

**Table 2:** **Weight Loss and Body Composition Outcomes**. Changes in body weight, fat mass, lean mass, visceral fat, and waist circumference as reported by the authors.

Author, Year	Weight Loss Duration	Δ % Weight	Δ Total Fat Mass (kg)	Δ % Lean Mass^a^	Δ % Visceral Fat	Δ Waist Circumference (cm)
Beavers et al., 2025	12 months	9.0–11.2 *p* < 0.05	−7.69 (95% CI −9.35, −6.03); *p* = 0.02	−1.9 *p* = 0.76	NR	NR
Bechtel et al., 2024	4–16 weeks (mean = 5 weeks)	5.5 (95% CI 7, 4); *p* < 0.05	NR	NR	NR	NR
			BL: 40.6 ± 10.5	BL: 61.7 ± 7.6 End: 60.5 ± 7.2 kg	BL: 3.1 ± 1.3 kg	BL: 117.0 ± 13.7
			End: 36.5 ± 10.4 *p* < 0.0001 within, *p* = 00.0028	*p* = 0.0016 within, *p* = 0.09	End: 2.7 ± 1.3 kg *p* = 0.0002 within, *p* = 0.01	End: 111.7 ± 14.2 *p* = 0.0003 within, *p* = 0.08
Justice et al., 2024	18 weeks	−11.3 ± 5.4 *p* < 0.001	NR	NR	NR	−10.4 ± 0.90 *p* < 0.0001
Dearborn et al., 2023	12 weeks	−3.0 ± 13.7 *p* = NS	NR	NR	NR	−5.0 ± 9.8; *p* = NS
Hamilton-Reeves et al., 2021	5–16 weeks (mean = 8.3 weeks	−5.4 (−5.55 kg) *p* = 0.0002 within, *p* = 0.01	−3.88 *p* = 0.001 within, *p* = 0.03	NR	NR	NR
				Non-bone LBM:	BL: 1993.5 ± 889.2 g	BL: 102.1 ± 8.8, End: 97.5 ± 8.7 *p* = 0.001 within, *p* = NS
				BL: 63.5 ± 6.6 kg	End: 1705.6 ± 834.4 g *p* = 0.003 within, *p* = NS	
				End: 62.8 ± 6.6 kg *p* = 0.01 within, *p* = NS		
Arterburn et al., 2019	16 weeks	MED 4&2&1: −5.0 *p* < 0.0001	MED 4&2&1: −3.75 ± 0.54 *p* < 0.0001	MED 4&2&1: −2.45 ± 0.43 *p* < 0.0001	MED 4&2&1: NR (−236 ± 47 g *p* < 0.0001)	MED 4&2&1: −5.2 ± 0.7 *p* = 0.0030
		OPT 5&1: −5.7 *p* < 0.0001	OPT 5&1: −4.53 ± 0.78 *p* < 0.0001	OPT 5&1: −1.49 ± 0.43 *p* < 0.05	OPT 5&1: NR (−239 ± 65 g *p* < 0.0001)	OPT 5&1: −6.2 ± 0.9 *p* = 0.0001
Beavers et al., 2019	24 weeks	−8.6 ± 0.4 *p* < 0.01	−7.1 [95% CI −8.1, −6.1]	<2% *p* = NS	NR	NR
			*p* = NR			
Serra et al., 2019	24 weeks	See Beavers 2019	See Beavers 2019	NR	−20.1	NR
					[BL: 2373 ± 1185 g	
					End: 1895 g (95% CI 1745–2045g)] *p* < 0.05 within, *p* < 0.01	
Normandin et al., 2018	22 weeks	−11.9 *p* < 0.001 within, *p* = NS	−7.9 ± 3.2 *p* < 0.0001 within, *p* = NS	−4.7^b^ [−2.3 ± 1.9 kg] *p* < 0.0001 within, *p* = NS	NR	−7.1 ± 7.2 *p* < 0.05 within, *p* = NS
Coleman et al., 2017	24 weeks^c^	Control: −15.7 ± 6.1	Both cohorts: −8–9 kg *p* = NR within, *p* = NS	−5 to 6 *p* = NR within, *p* = NS	NR	Control: 15.7 ± 6.7
		D/HBG: −13.9 ± 7.0				D/HBG: 10.1 ± 6.8 *p* < 0.0001 within for both, *p* = 0.027
		*p* < 0.0001 within for both, *p* = NS				
Moldovan et al., 2016	12 weeks	−8.8 *p* = 0.000 within, *p* = 0.028	NR	−2.5 *p* = 0.001 within, *p* = NS	NR	NR
			BL: 58.3 ± 17.5 End: 49.3 ± 18.6 *p* = 0.000 within, *p* = 0.019			
Coleman et al., 2015^d^	12 weeks^c^	−10.1	−10.9 ± 5.6 *p* < 0.0001 within	NR	NR	−9.8 ± 5.9 *p* < 0.0001 within
		*p* < 0.0001 within		Reported as <5 through 24 weeks *p* < 0.0001 within		
Kiel et al., 2015^d^	12 weeks^c^	−8.6	−9.6 ± 3.7 *p* = 0.002	−3.2 *p* = 0.023	NR	−9.0 ± 4.7 *p* < 0.01
		*p* < 0.0001 within				
Shikany et al., 2013	26 weeks	−6.7 *p* < 0.05 within, *p* = 0.0002	−6.4 *p* < 0.05 within, *p* = 0.0011	FFM: −2.25^b^ [BL: 58.6 ± 11.3 End: 57.4 ± 10.2 Δ −1.2] *p* < 0.05 within, *p* = 0.0162	NR	−5.7 *p* < 0.05 within, *p* = 0.0064
Coleman et al., 2012^d^	Final Visit (Mean: 19.6 ± 13.5 weeks)^c^	−12.0 ± 7.1 *p* ≤ 0.0001	NR	NR	NR	BL: 46.5 ± 6.0 in.
			BL: 93.2 ± 28.3 lbs. End: 69.5 ± 26.5 lbs. *p* ≤ 0.0001	BL: 114.4 ± 22.8 lbs. End: 110.3 ± 21.8 lbs. *p* ≤ 0.0001		End: 41.8 ± 5.7 in. *p* ≤ 0.0001
Davis et al., 2010	16 weeks	−12.3	NR	NR	NR	−13.0
		*p* < 0.0001 within, *p* = 0.001	Reported as −5.6% absolute reduction in body fat *p* < 0.0001	BL: 56.8 ± 11.4 End: 55.0 ± 10.4 *p* < 0.0001 within, *p* = NR	Reported as VFR (Score 0–59)	*p* < 0.0001 within, *p* = 0.003
					Baseline: 13.8 ± 3.8	
					End: 10.6 ± 3.5	
					Δ −3.2 (−25.4%)	
					*p* < 0.0001 within and between	

**Note:***p* values represent between-group comparisons unless otherwise noted (i.e., *p* values with “within” listed afterwards signify within-group changes). NR = not reported; NS = not significant (*p* > 0.05); BL = baseline; End = end of weight loss intervention; MED: Medifast 4&2&1 Plan, OPT: 5&1 Plan [28]; Control = without diabetes/high blood sugar [26]; D/HBG = diabetes/high blood sugar; LBM: lean body mass; FFM = fat-free mass; VFR = visceral fat rating.

a Lean mass/lean body mass reflect DXA or BIA-derived values unless otherwise specified.

b Calculated by taking reported change/baseline x 100.

c Primary endpoint as specified by authors.

d Between-group comparisons not applicable for single-arm chart reviews.

### 3.2 Body composition

Body composition outcomes are summarized in Table 2. Total fat mass reductions ranged from 3.2 to 11 kg across trials [21,24,25,28,36]. Six RCTs utilizing a non-Medifast meal plan control and 1 non-randomized open-label pilot trial reported significant between-group reductions in total fat mass (*p* < 0.05) [21,22,24,25,27,28,30]. Three chart reviews and Normandin et al., 2018 (an RCT with a Medifast meal plan as the control) reported significant within-group reductions (*p* < 0.05) [[18], [19], [20],^23^,^30^].

Waist circumference decreased by approximately 5–16 cm across 8 RCTs and 3 chart reviews [[18], [19], [20],[23], [24], [25],^27^]. Among the 7 RCTs using a non-Medifast meal plan control, 5 reported significant between-group reductions, while the remaining RCT and 3 chart reviews reported significant within-group reductions (*p* < 0.05). Visceral fat outcomes were reported in 4 RCTs and 1 non-randomized open-label pilot study. All 4 RCTs reported statistically significant between-group reductions, while the open-label pilot trial (n = 20) did not reach statistical significance for between-group differences. [24,27,28,30,37]. Serra et al. reported the largest reduction in visceral fat of 20.1% at 24 weeks [37].

Losses in lean mass or fat-free mass were present but comparatively modest. In both older adults and general adult populations, reductions in lean mass were typically less than 2 kg or less than 5% from baseline, including studies where total weight loss exceeded 8%–10% [18,19,[21], [22], [23], [24], [25],^27^,^28^,^30^,^36^].

### 3.3 Cardiometabolic outcomes

The impact of weight loss interventions using Medifast meal plans on cardiometabolic risk factors, including blood pressure, lipid profiles, and markers of glycemic control, is summarized in Table 3.

**Table 3:** **Cardiometabolic Outcomes**. Changes in blood pressure, blood lipid profiles, and glycemic markers as reported by the authors.

Author, Year	Duration	Δ Blood Pressure (mmHg) (SBP/DBP)	Δ Total Cholesterol (mg/dL)	Δ LDL-C (mg/dL)	Δ HDL-C (mg/dL)	Δ Triglycerides (mg/dL)	Δ Fasting Glucose (mg/dL)	Δ Fasting Insulin (μIU/mL)	Δ A1c (%)
Bechtel et al., 2024	4–16 weeks (mean = 5 weeks)	NR	NR	NR	NR	NR	NR	NR	NR
			BL: 168.45 ± 33.65	BL: 98.42 ± 28.05	BL: 42.25 ± 11.59	BL: 154.7 ± 118.68	BL: 103.05 ± 16.63 End: 100.3 ± 18.11 *p* = NS within and between	BL: 10.81 ± 6.19	
			End: 143.0 ± 26.46 *p* < 0.0001 within and between	End: 76.95 ± 21.40 *p* < 0.0001 within, *p* = 0.0002	End: 43.45 ± 11.21 *p* = NS within and between	End: 121.6 ± 127.09 *p* = 0.0346 within, *p* = NS		End: 7.49 ± 3.37 *p* = 0.0012 within, *p* = 0.0441	
Justice et al., 2024	18 weeks	−8.2 ± 2.8 *p=*0.21/−4.6 ± 1.8 *p* = 0.03	−11.8 ± 4.5 *p* = NS	−5.3 ± 4.0 *p* = NS	Reported as *p* = NS (data NR)	Reported as *p* = NS (data NR)	NR	−5.00 ± 2.57 *p* = 0.03	−0.2% ± 0.03 *p* < 0.0001
Dearborn et al., 2023	12 weeks	+1 ± 26 SD; *p* = NS/+2 ± 18; *p* = NS	NR	NR	NR	NR	NR	NR	NR
Hamilton-Reeves et al., 2021	5–16 weeks (mean = 8.3 weeks)	NR	NR	NR	NR	NR	NR	NR	NR
		Reported as: SBP decreased BL to end (*p* = 0.02 within; *p* = NS	BL: 185.6 ± 37.7	BL: 107.6 ± 28.8	BL: 53.2 ± 14.2	BL: 132.3 ± 154.6	BL: 108.8 ± 26.9	BL: 8.8 ± 7.0	
			End: 177.6 ± 47.8 *p* = NS within and between	End: 104.2 ± 38.6 *p* = NS within and between	End: 54.7 ± 14.0 *p* = NS within and between	End: 92.8 ± 35.7 *p* = NS within and between	End: 98.6 ± 15.9 *p* = NS within and between	End: 5.5 ± 2.7 *p* = 0.03 within, *p* = NS	
Serra et al., 2019	24 weeks	NR	NR	NR	NR	NR	NR	NR	NR
				BL: 109.6 ± 34.1	BL: 51.2 ± 15.2	BL: 135.7 ± 64.0	BL: 106.5 ± 16.2	BL: 22.3 ± 12.8	
				End: 105.22 (95% CI 97.76, 112.68)] *p* = NS within and between	End: 53.88 (95% CI 50.97, 56.79)] *p* = NS within, *p* < 0.01	End: 105.79 (95% CI 92.31–119.27) *p* < 0.05 within, *p* < 0.01	End: 102.77 (95% CI 98.87–106.68) *p* < 0.05 within and between	End: 14.93 (95% CI 12.30–17.55) *p* < 0.05 within, *p* < 0.01	
Shaver et al., 2018	24 weeks	SBP: −7.46 (95% CI −11.30, −3.62) *p* = NS	NR	NR	NR	NR	NR	NR	See Serra 2019
		DBP: NR							
Coleman et al., 2017	24 weeks^a^	Control: −11.4 ± 17.6/−6.7 ± 12.3; D/HBG: −19.6 ± 21.9/−8.5 ± 12.1 *p* < 0.0001 within both cohorts, *p* = NS	NR	NR	NR	NR	NR	NR	NR
Coleman et al., 2015^b^	12 weeks^a^	−11.3 ± 16.7/−6.6 ± 12.6 *p* < 0.0001 within for both	NR	NR	NR	NR	NR	NR	NR
Kiel et al., 2015^b^	12 weeks^a^	−17.7 ± 17.6/−8.4 ± 8.0 *p* = 0.008/*p* = 0.003 within	NR	NR	NR	NR	NR	NR	NR
Shikany et al., 2013	26 weeks	−3.2/−1.6	−8.4 *p* < 0.05 within, *p* = 0.0355	−9.2 *p* < 0.05 within, *p* = 0.0119	+1.2 *p* = NS within and between	−3.7 *p* = NS	−1.1 *p* = NS within and between	NR	NR
		*p* = NS within and between							
Coleman et al., 2012^b^	Final Visit (Mean: 19.6 ± 13.5 weeks^a^)	−8.8/−6.0	NR	NR	NR	NR	NR	NR	NR
		*p* ≤ 0.0001 within							
Davis et al., 2010	16 weeks	NR	NR	NR	NR	NR	NR	NR	NR
		SBP:	BL: 191.2 ± 39.5	BL: 117.3 ± 31.8	BL: 52.0 ± 16.0	BL: 109.2 ± 63.7			
		BL:125.4 ± 13.9	End: 181.3 ± 40.4 *p* = NS within and between	End: 111.4 ± 34.4 *p* = NS within and between	End: 51.6 ± 11.3 *p* = NS within and between	End: 91.8 ± 52.7 *p* = NS within and between			
		End: 113.6 ± 14.3 *p* < 0.0001 within							
		DBP:							
		BL: 83.2 ± 9.5 End: 74.2 ± 8.8							
		DBP *p* = 0.001 within							
		*p* = NS							

**Note:***p* values represent between-group comparisons unless otherwise noted (i.e., *p* values with “within” listed afterwards signify within-group changes). NR = not reported; NS = not significant (*p* > 0.05); BL = baseline; End = end of weight loss intervention; Control = without diabetes/high blood sugar [26]; D/HBG = diabetes/high blood sugar.

a Primary endpoint as specified by authors.

b Between-group comparisons not applicable for single-arm chart reviews.

#### 3.3.1 Blood pressure

Blood pressure outcomes were heterogeneous. Single-arm retrospective chart reviews reported within-group reductions in systolic blood pressure (SBP: 8.8–19.6 mmHg) and diastolic blood pressure (DBP: 6.0–8.5 mmHg), though with large standard deviations (SBP: ±16.7 to ±21.9 mmHg; DBP: SD ± 8.0 to ±12.6 mmHg) reflecting substantial individual variability [[18], [19], [20],^26^]. In RCTs, findings varied. Davis et al. observed significant within-group reductions (SBP: 11.8 mmHg, *p* < 0.0001; DBP: 9.0 mmHg, *p* = 0.001) at 16 weeks, though between-group differences versus control were non-significant [24]. Other RCTs reported smaller or non-significant changes [25,31], with one pilot trial showing slight increases [35].

#### 3.3.2 Lipids

Blood lipid profile changes were variable. Total cholesterol (TC) and LDL-cholesterol (LDL-C) showed between-group significance in 2 of 5 [25], [27], [2] of 6 studies [25,27], respectively (Table 3). Significant decreases were observed by Bechtel et al. (TC: baseline = 168.45 ± 33.65, end = 143.0 ± 26.46 mg/dL, *p* < 0.0001 within-group and between-group; LDL-C: baseline = 98.42 ± 28.05, end = 76.95 ± 21.40, *p* < 0.0001 within-group, *p* = 0.0002 between-groups) and Shikany et al. (TC: 8.4 mg/dL, *p* = 0.0355 between-groups; LDL-C: 9.2 mg/dL, *p* = 0.0119 between-groups) [25,27]. HDL-cholesterol changes were modest (Table 3), with between-group significance reached only by Serra et al. [37]. Triglyceride reductions were generally non-significant (Table 3), with 2 of 6 studies reporting within-group significance [27,37] and one reporting between-group significance [37].

#### 3.3.3 Glycemic control

Four studies reported on fasting glucose and/or fasting insulin [25,27,29,30,37]; one study reported changes in A1c [29] (Table 3). Three of 4 studies showed non-significant between-group changes in fasting glucose [27,29,30,37], with one study, Serra et al., reporting significance (baseline = 106.5 ± 16.2, end = 102.77 mg/dL (95% CI 98.87–106.68) *p* < 0.05 within-group and between-group) [37]. Three of 4 studies reported significant between-group reductions for fasting insulin [27,29,37]; the open-label pilot trial showed non-significant results [30]. Justice et al. observed an A1c reduction of 0.2% (*p* < 0.0001) in adults with prediabetes over 18 weeks [29].

## 4 Discussion

This qualitative narrative review synthesized evidence from 17 publications to evaluate the impact of Medifast's structured higher protein meal replacement plans on weight and metabolic health. The findings demonstrate that these interventions facilitate clinically meaningful short-term weight loss, typically ranging from 5% to 12%, across varied adult populations living with overweight/obesity, including those with coexisting conditions and older adults. Beyond total weight reduction, a defining characteristic of these outcomes was the quality of weight loss, characterized by significant reductions in visceral adiposity alongside the preservation of lean body mass, relative to the amount of weight lost. While improvements in glycemic markers were generally favorable, data was limited. Blood pressure and lipid outcomes were variable and generally non-significant. Collectively, these data provide a framework for understanding how structured higher protein meal replacement plans and behavior modification can serve as an effective dietary foundation within a comprehensive obesity care model.

### 4.1 Weight loss

Across the reviewed literature, weight loss interventions utilizing Medifast meal plans facilitated clinically meaningful short-term weight loss in adults living with overweight or obesity, typically meeting or exceeding the ≥5% threshold established by clinical guidelines for cardiometabolic benefit [4,5,7,8,[38], [39], [40]]. These findings align with broader evidence on partial meal replacement interventions, which are recognized as an evidence-based strategy for weight and metabolic improvement [4,7]. Even within the evolving landscape of obesity management, which now includes highly effective pharmacotherapies, structured dietary approaches such as partial meal replacement models remain a relevant, non-pharmacologic option that can complement pharmacotherapy. Current clinical guidelines continue to support their use as part of a comprehensive, individualized treatment strategy, particularly for patients seeking or requiring alternatives or supplemental approaches to medication or surgery [4,7,8].

Systematic reviews indicate that meal replacement programs can achieve modestly greater short-term weight loss compared to standard food-based advice [41] and at least 4% greater loss than counseling alone [42]. Arterburn et al., an RCT included in this review, found that participants using Medifast meal plans that included coaching and community support achieved 5.0%–5.7% weight loss at 16 weeks, significantly exceeding that of a self-directed reduced-calorie control diet [28].

### 4.2 Body composition and lean mass preservation

The observed body composition changes demonstrate a favorable pattern of fat mass reduction, including consistent decreases in waist circumference and visceral adiposity, while lean mass losses remained modest. While these results are encouraging, the magnitude of lean mass loss is generally proportional to the total weight reduction achieved [43,44]. Consequently, the moderate weight loss achieved in these trials will likely result in lower lean mass loss compared to other more intensive weight loss modalities (e.g., bariatric surgery, highly effective obesity medications).

Notably, three of the included randomized controlled trials were conducted in older adults (60–85 years), a population at heightened risk for sarcopenia and functional decline [21,23,36]. This is clinically relevant because central adiposity drives cardiometabolic risk and mortality independent of BMI [11,[45], [46], [47]]. Preserving lean mass is a critical goal during weight loss to maintain metabolic rate and physical function, particularly in older adults at risk for sarcopenia [48,49].

The relatively high complete protein content of Medifast meal plans (80–170 g/day) likely supports lean mass retention, aligning with existing evidence that suggests higher protein intake during energy restriction helps maintain lean mass [48,50,51]. While exercise was encouraged but not standardized in most trials, pairing these dietary interventions with resistance training remains a best practice for optimizing functional outcomes [52].

### 4.3 Cardiometabolic outcomes

The clinical value of weight loss depends largely on its impact on cardiometabolic risk factors [5]. Improvements in glycemic markers (fasting glucose, insulin, and A1c) were the most consistent across the reviewed studies, with significant within-group and between-group reductions observed [27,29,30,37]. Even modest reductions in these markers are associated with a lower risk of type 2 diabetes and cardiovascular disease [53,54].

In contrast, blood pressure and lipid outcomes were heterogeneous. Chart reviews reported systolic blood pressure reductions of 8–20 mmHg [[18], [19], [20],^26^], magnitudes associated with significant cardiovascular risk reduction [9,55], while RCT results typically did not show significant between-group differences when compared to standard reduced-calorie food-based diets [24,25,31]. Similarly, lipid changes were less consistent, with triglycerides generally non-significant and TC and LDL-C showing variable responses [24,25,27,30,37]. The observed variability in cardiometabolic outcomes may be influenced by differences in baseline participant characteristics across the included studies. These findings are consistent with existing literature, which often demonstrates heterogeneous results for blood pressure and lipid markers following non-surgical weight loss interventions [56,57].

Overall, the observed improvements in cardiometabolic outcomes are consistent with evidence that even modest weight loss can yield clinically meaningful benefits [39,40]. However, these changes are likely attributable to the broader physiological effects of weight loss rather than unique effects of the intervention itself, as reductions in blood pressure, lipids, and glycemic markers are well-established responses to caloric restriction and weight loss [39,40].

### 4.4 Plausible mechanisms and contextual contributors

Mechanistically, weight loss interventions utilizing Medifast meal plans likely act through multiple pathways beyond macronutrient composition alone. By providing defined energy targets and portion control, the structured format reduces the decision burden and self-regulation challenges inherent in modern obesogenic environments [17]. Additionally, micronutrient fortification of meal replacements may help mitigate nutritional gaps during intensive calorie restriction, a feature that may be particularly valuable in routine practice settings as delivering more personalized, comprehensive and ongoing dietary counseling is resource-intensive [38]. Behavioral support, such as individual coaching, group support, and structured lifestyle curricula, can also play a key role in outcomes, even though the specific combination of these modalities varied across the reviewed studies.

It is also important to note that the observed reductions in body composition and cardiometabolic metrics are largely consistent with known physiological effects of weight loss in general. Thus, while program structure, behavioral support, and nutritional composition may contribute to adherence, implementation, and outcomes, improvements are also driven in part by caloric restriction and weight loss itself. Accordingly, these findings should be interpreted as reflective of both the intervention framework and the broader metabolic effects of weight loss.

### 4.5 Safety and adherence

Weight loss interventions utilizing Medifast meal plans appear generally well-tolerated in the short term, with self-reported adverse events (e.g., hunger, fatigue, constipation) typical of calorie restriction and no reported serious events directly attributed to the plans [21,24,25,28]. While the structured nature of the program may facilitate adherence for many, real-world completion rates may vary [19,26]. Systematic collection of adherence data and reasons for discontinuation would strengthen future evaluations.

## 5 Limitations

This review has several limitations that affect the interpretation of the findings. First, the evidence base consists exclusively of studies examining weight loss interventions that utilize Medifast meal plans. Direct comparison to other commercial meal replacement programs (e.g., OPTIFAST, Nutrisystem) or other evidence-based dietary approaches (e.g., Mediterranean diet, DASH diet, intensive food-based counseling) was not an objective of this review. This limits the ability to assess whether outcomes are specific to Medifast meal plans or are generalizable to other commercial higher protein meal replacement approaches more broadly.

Second, study quality and design were variable. The RCTs in the review had modest sample sizes (n = 34–198) and some lacked true dietary control groups [21,23,36]. The impact of small sample sizes is particularly evident in pilot data where individual outliers, such as a single participant experiencing significant weight gain, can disproportionately skew aggregate outcomes and potentially mask the broader trend of clinical benefit [35]. The inclusion of retrospective chart reviews introduces potential selection bias, as these cohorts only include individuals who were actively engaged with the program. Additionally, the program-specific nature of the evidence base introduces the potential for bias related to study design, sponsorship, and reporting.

Given the heterogeneity in study cohorts, interventions, and outcome measures, this review employed a narrative qualitative evidence synthesis rather than a formal meta-analysis. Our inclusion criteria aimed to comprehensively capture all peer-reviewed publications utilizing Medifast meal plans as weight loss interventions, minimizing selection bias in the review process. However, the narrative approach and inclusion of retrospective chart reviews introduce inherent limitations, including potential publication bias and selection or reporting biases within the original studies. These factors should be considered when interpreting the findings.

Third, the relatively short duration of most included interventions (4–26 weeks) limits conclusions regarding long-term durability of weight and metabolic health outcomes. While some of the included studies (n = 6) had weight maintenance phases, evaluation of those outcomes was outside the scope of this review. Consistent with other dietary approaches, the benefits of weight loss interventions utilizing meal replacements are often subject to weight regain if the structured plans or behavioral supports are withdrawn [42].

Finally, the variation in behavioral components, such as coaching intensity and lifestyle curricula, alongside non-standardized physical activity levels make it difficult to isolate the specific effects of the meal plans from the broader programmatic context. However, it is important to note that these elements are integral to the comprehensive nutrition and lifestyle framework in which these meal plans are intended to be used.

## 6 Clinical considerations

Medifast meal plans represent one commercially available meal replacement option for weight loss and metabolic health interventions. Successful implementation requires integration within a four-pillar obesity care framework where weight loss interventions utilizing Medifast meal plans serve as the dietary foundation. Physical activity, including regular aerobic and especially resistance training (to help retain lean mass during weight loss) would also be a key pillar; this would continue to be of critical importance for long-term weight loss maintenance as well. Behavioral support, including the support of a coach, counselor, or trained facilitator, would likely improve outcomes [28]. Behavioral modification emphasizes development of healthy eating behaviors, self-monitoring, stress management, sleep hygiene and other key areas of health and well-being are also critical to holistic health and well-being. Where clinically indicated, medical intervention, such as obesity pharmacotherapy may also be warranted [4,58].

Weight loss interventions utilizing Medifast meal plans may be considered for:

1. Adults living with overweight or obesity (BMI ≥25 kg/m^2^).
2. Individuals who prefer structure, convenience, reduced meal preparation and decision-making burden.
3. Patients with obesity-related comorbidities or metabolic syndrome components (i.e., metabolic dysfunction) where initial weight loss is a treatment goal.

Conversely, they may not be appropriate for:

1. Those who are pregnant or lactating.
2. Patients with advanced chronic kidney disease or other conditions who require specific protein restrictions.
3. Individuals with strong preference for whole-food approaches.

Out-of-pocket cost of meal replacements is another key consideration for clinicians when working with patients. However, when integrated into a comprehensive care plan, these costs may be partially offset by reduced expenditures on conventional groceries.

Medical supervision is recommended, particularly for individuals with co‐morbidities and those taking medications that may require adjustment during weight loss (e.g., diabetes medications, antihypertensives, anticoagulants).

Clinicians should frame weight loss interventions, like Medifast's comprehensive nutrition and lifestyle program and meal plans, as a tool for chronic disease management and set realistic expectations: weight regain is common, and successful maintenance requires indefinite behavioral vigilance, continued use of program tools (e.g., select meal replacements, self-monitoring, physical activity, habit tracking) and ongoing support [58]. The strategic use of partial meal replacement models for long-term weight management is supported by obesity clinical guidelines as an effective modality for weight stability [4,7,8]. Therefore, utilizing a comprehensive system that includes structured weight maintenance support is recommended.

As mentioned previously, this review examined only studies with weight loss interventions that utilized Medifast meal plans. Clinicians should consider multiple evidence-based options when recommending weight loss and metabolic health strategies, individualized to patient preferences, comorbidities, and resources.

## 7 Conclusions

This narrative qualitative evidence review synthesized published evidence on weight loss interventions utilizing Medifast meal plans (5&1, 4&2&1, 5&2&2) for weight loss and metabolic health in adults with overweight or obesity. Across heterogeneous study designs including randomized controlled trials, retrospective chart reviews, and secondary data analyses, Medifast's comprehensive nutrition and lifestyle program and meal plans were associated with clinically meaningful short-term weight loss, typically achieving 5%–12% reductions in body weight over 4–26 weeks. Weight loss was accompanied by favorable body composition changes including significant reductions in fat mass and visceral fat mass across majority of studies, alongside a modest loss of lean mass. Clinically meaningful improvements in some cardiometabolic risk factors, particularly glycemic markers, were observed, however findings for blood pressure and lipids were variable and often not significant compared to standard reduced-energy food-based controls in RCTs.

Future studies should evaluate long-term weight maintenance strategies and durability of cardiometabolic improvements for weight loss interventions utilizing Medifast meal plans. Additionally, assessing impacts on bone health, inflammatory markers, and other body composition and cardiometabolic markers, would strengthen the evidence base.

## 8 Key takeaways for clinicians

1. **Efficacy:** Weight loss interventions utilizing Medifast's comprehensive nutrition and lifestyle program and meal plans consistently produced clinically meaningful short-term weight loss, with most studies achieving ≥5% within six months, meeting guideline thresholds for cardiometabolic benefit.
2. **Body Composition:** Favorable changes including reductions in total and visceral fat with lean mass retention suggest that higher complete protein meal replacement plans may support quality weight loss and body composition changes.
3. **Comprehensive Care Integration:** Weight loss interventions utilizing Medifast's comprehensive nutrition and lifestyle program and meal plans are best integrated as one component of the four-pillar obesity care framework (nutrition, physical activity, behavioral modification, medical interventions). Success depends on thoughtful patient selection, integration of meal plans with a comprehensive nutrition and lifestyle system, and intentional weight loss maintenance planning and follow-up to support effectiveness and long-term sustainability.

## CRediT author statement

AM: Conceptualization, Methodology, Investigation, Data Curation, Writing - Original Draft, Writing - Review & Editing. CC: Methodology, Investigation, Data Curation, Writing - Review & Editing. JK: Methodology, Supervision, Writing - Review & Editing. KB: Methodology, Writing - Review & Editing. SJ: Conceptualization, Resources, Supervision, Writing - Review & Editing.

All authors have reviewed and approved the final manuscript.

## Ethical review

This submission is a narrative qualitative evidence review of previously published research and did not involve new interventions affecting human subjects. All original studies included in this review were conducted in accordance with the Declaration of Helsinki and received approval from their respective Institutional Review Boards or Ethics Committees. Informed consent was obtained from all participants in the original studies as reported in the primary publications.

## Declaration of artificial intelligence (AI) and AI-assisted technologies

During the preparation of this work, the authors used Abacus. AI (Gemini 3 Flash) for the purpose of editing and refining language to improve the clarity and readability of the manuscript. AI was not used for data collection, data analysis, the interpretation of results, or the generation of original scientific content. After using this tool, the authors reviewed and edited the content as needed and take full responsibility for the content of the publication.

## Source of funding

This work was supported by Medifast, Inc. [no grant number applicable]. Medifast, Inc. was involved in the study design and the collection, analysis, and interpretation of data conducted for this qualitative narrative review; this also includes the writing of and the decision to submit the manuscript for publication.

## Declaration of competing interests

Alexandra Miller, Christopher Coleman, Jessica Kiel, and Satya Jonnalagadda are employees of Medifast, Inc. Kristen Beavers has received research funding and/or honoraria from Medifast, Inc. for her role as a consultant and investigator.
